# 3DAD: Super-Resolution Image Synthesis from Anisotropic CT Images Using a Three-Dimensional Adversarial Diffusion Model

**DOI:** 10.3390/bioengineering13060595

**Published:** 2026-05-22

**Authors:** Jianliang Lu, Ho Ming Cheng, Benjamin Xin Hao Fang, Chun On Anderson Tsang, Sarah Yu, Wai-Kay Seto, Philip Leung Ho Yu, Keith Wan-Hang Chiu

**Affiliations:** 1 Department of Medicine, School of Clinical Medicine, The University of Hong Kong, Hong Kong; 2 Department of Diagnostic & Interventional Radiology, Hong Kong Sanatorium & Hospital, Hong Kong; 3 Wessex Neurological Centre, Southampton General Hospital, University of Southampton, Southampton SO16 6YD, UK; 4 Department of Medicine, The University of Hong Kong-Shenzhen Hospital, Shenzhen 518000, China; 5 State Key Laboratory of Liver Research, The University of Hong Kong, Hong Kong; 6 Department of Mathematics and Information Technology, The Education University of Hong Kong, Hong Kong; 7 Department of Computer Science, The University of Hong Kong, Hong Kong; 8 Department of Diagnostic and Interventional Radiology, Queen Elizabeth Hospital, Hong Kong

**Keywords:** computed tomography (CT), thin slice, thick slice, synthetic, diffusion model

## Abstract

High-resolution thin-slice computed tomography (CT) images are often compressed into lower-quality thick-slice images for long-term storage, necessitating synthesis for medical diagnosis. In this paper, we propose a novel 3D adversarial diffusion model (3DAD) for high-fidelity synthesis of thin-slice CT from compressed thick-slice CT. 3DAD is composed of a generator and a discriminator for synthesizing denoised thin-slice images from random noise and source images and distinguishing between noised samples from real and denoised synthetic thin-slice images. Specific models were trained on two-slice to six-slice scenarios for abdominal data, using thick-slice CT compressed from real thin-slice CT as the source. 3DAD was evaluated at the time of HCC diagnosis, at the observation and patient levels, using real thin-slice and synthetic thin-slice CT, with DeLong’s test to compare the similarity of receiver operating characteristic (ROC) curves. We further evaluated 3DAD on real-world data with both thin and thick images, with the synthetic image quality assessed by radiologists and in radiomics feature analysis. Based on the external dataset with 548 samples, the achieved mean squared error (MSE), peak signal-to-noise ratio (PSNR), and structural similarity index measure (SSIM) values were 81.374, 29.478, and 0.916, respectively, for the five-slice scenarios at the portal venous phase. The Areas Under Curves (AUCs) achieved were 0.896 on synthetic thin-slice images compared with 0.889 on real thin-slice images at the observation level (*p* = 0.028) and 0.854 versus 0.846, correspondingly, at the patient level (*p* = 0.055). For evaluation on the real-world testing dataset after fine-tuning at the portal venous phase, the MSE, PSNR, and SSIM were 70.435, 30.243, and 0.94, respectively. Radiologist evaluation confirmed the high quality of the synthetic image, with no significant difference in the majority of cases across all five parameters, except for radiologist 2, in realistic and consistent situations, under which at least 41 of 43 synthetic images were assessed as equal to or above grade 3. Our 3DAD enabled the synthesis of thick-slice CT images into high-resolution thin-slice images, facilitating high-fidelity volume image application in HCC diagnosis.

## 1. Introduction

A sharp increase in the utilization of medical images in patient management, particularly advanced cross-sectional imaging modalities, has led to a large accumulation of medical images, such as computed tomography (CT). There were approximately 74 million CT procedures in the United States in 2016, accounting for about 18% of the world’s estimated total [1]. High-resolution thin-slice CT images with a thickness of 1.25 mm or less are recommended for clinical diagnosis [2,3,4]. Huge thin-slice three-dimensional CT images require a lot of storage, which leads to the compression of thin-slice images into lower-quality thick-slice images in Picture Archiving and Communication Systems (PACSs) for long-term storage, accompanied by a decrease in spatial resolution, making the reuse of these images in clinical diagnosis and medical research difficult [5]. Furthermore, thick-slice CT with a 5 mm thickness remains prevalent in many developing countries, even though scanners can acquire thin slices, increasing the risk of misdiagnosis and its consequences [6]. As artificial intelligence (AI) demonstrates its power in medical applications and moves towards building foundation models for generalist medical AI, massive, high-quality training datasets are needed. In addition, driven-up costs associated with data collection have made the reversal of the thick-slice images back into high-resolution thin-slice images necessary, allowing comparable capability in clinic applications between synthetic and real thin-slice images.

Deep convolutional neural network (CNN) models show significant power in image recognition once introduced [7,8]. Pix2Pix was introduced for image-to-image translation with conditional generative adversarial networks (GANs) on 2D natural images, and it was soon applied to medical applications for diverse target medical image translation [9,10,11]. Impressive performance in medical image translation based on the Pix2Pix model was soon surpassed by a diffusion-based model [12]. SynDiff was used for 2D unsupervised medical image translation with adversarial diffusion models, which introduced a cycle-consistent architecture to enable training on unpaired datasets [13]. These models are restricted to 2D images, while 3D images are frequently used for complex diagnostic decision-making. VTS was the first model used for 3D thin-slice generation with a conditional GAN [14], but the chosen Unet-based GAN architecture limited its performance. Zolnamar reported a 3D denoising diffusion probabilistic model (3D-DDPM) that enables 3D brain tumor image synthesis, but it is not useful for whole-3D-image generation, as it is limited by the training time caused by the short diffusion steps [15]. Researchers from Google released another method for reducing the storage of 3D CT images by extracting the input CT volume into an information-rich embedding that can be used for rapid training. The limitation of this strategy is the black-box AI model used, which limits the visibility of image embeddings [16]. Although many deep learning models have been developed to build super-resolution medical images, little attention has been paid to synthesizing thin-slice CT, especially to validate synthetic thin-slice images in clinical applications comprehensively.

Diffusion models have the potential to achieve state-of-the-art performance in image generation, but they suffer from intensive computation, which is time-consuming and GPU-intensive due to a large number of diffusion steps. We propose a novel 3D adversarial diffusion model (3DAD) that applies faster sampling with a larger step size and fewer diffusion steps, enabling the high-fidelity synthesis of thin slices from compressed thick slices [13,17,18,19,20]. The purpose of this multicenter study was to develop a deep learning model to generate thin slices (≤1.25 mm) from thick slices (≥5 mm) and to assess the potential of synthetic thin-slice images for clinical application quantitatively and qualitatively. We explored whether synthetic thin-slice images can achieve performance comparable to that of radiologist-reviewed images and maintain comparable quality after review in liver cancer diagnosis.

## 2. Method

### 2.1. Abdomen Base Dataset

**Figure 1:**
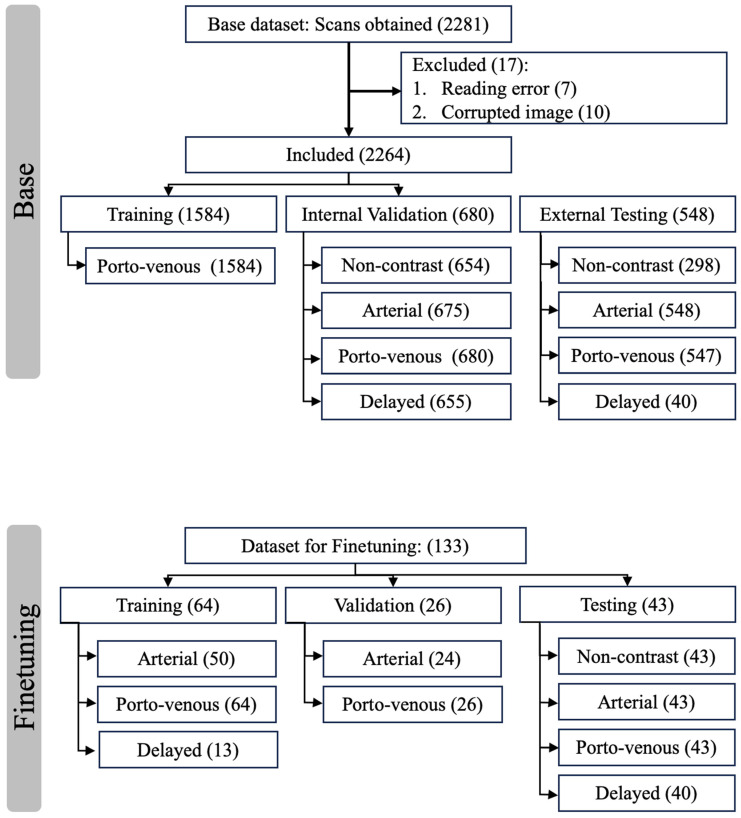
The patient selection process for the abdomen base and fine-tuning dataset.

For the abdomen base dataset, the internal cohort with 2281 patients was collected from archived thin-slice multi-phase CT liver images scanned from October 2008 to August 2020 from six medical centers in our locality (The University of Hong Kong, Queen Mary Hospital, The University of Hong Kong-Shenzhen Hospital, Pamela Youde Nethersole Eastern Hospital, Queen Elizabeth Hospital, Kwong Wah Hospital) (Figure 1). To evaluate HCC diagnosis performance, all CT scans with at least one untreated liver observation with a size of at least 5 mm were included. All scans included were required to have a slice thickness ≤ 1.25 mm and were performed in Asian individuals aged 18+ years. Scans performed after loco-regional therapy, including thermal ablation, transarterial chemoembolization or radioembolization, and external beam radiation therapy, were excluded. Incomplete scans and scans with reading errors upon loading the DICOM files were also excluded. Another external cohort of 548 cases was collected independently from Sun Yat-Sen University Cancer Center during 2013–2018 using the same criteria. All four phases were included, and all CT scans in the internal and external cohorts were typically extended from the lung base to the iliac crest and segmented with TotalSegmentor [21].

### 2.2. Abdomen Fine-Tuning Dataset

To evaluate the performance of 3DAD on real-world thick-slice CT, we collected a fine-tuning dataset including 133 patients with thin- (≤1.25 mm) and thick-slice (5 mm) CT pairs from the Queen Elizabeth Hospital (1 mm thin vs. 5 mm thick) and the medical center of the University of Hong Kong (1.25 mm thin vs. 5 mm thick) from November 2022 to January 2023 (Figure 1). Similarly, all CT scans included had at least one untreated liver observation with a size of at least 5 mm. Non-contrast, late hepatic arterial, portal-venous, and delayed-phase images were included using the same criteria.

### 2.3. Model Training

**Figure 2:**
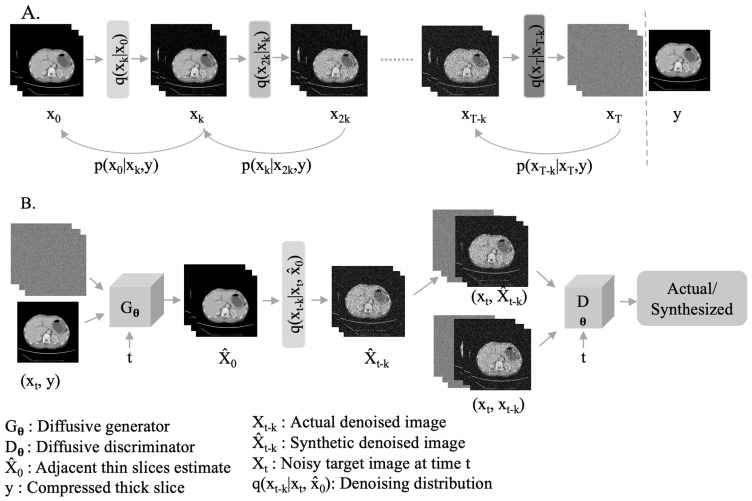
The architecture of the 3D adversarial diffusion model. (**A**). Forward and reverse processes of the diffusion model with step size k ≫ 1. (**B**). Diffusion generator and discriminator. Every n (n = 2, 3, 4, 5, 6) thin slice in the real thin-slice 3D CT images was compressed into 1 thick slice and synthesized to the original number of slices with a diffusion generator.

The developed novel 3DAD model for super-resolution CT image synthesis comprises a generator and a discriminator (Figure 2, Supplementary Method S1 for details). The diffusion generator synthesized a denoised thin-slice image given a random noise and a thick-slice image as the source. The diffusion discriminator distinguished between noisy samples from real thin-slice images and denoised synthetic thin-slice images. A residual module equipped with a three-dimensional Unet acted as the backbone of the generator in 3DAD. Each residual module included two subblocks, and six such modules were used in both the encoder and decoder of the Unet. The residual module also received a temporal embedding and a 256-dimensional random latent. An adversarial loss, together with an L1 loss, was used for the generator. The discriminator in 3DAD used six blocks, each with two convolutional layers. Each discriminator block also received the temporal embedding. The binary cross-entropy loss was used for the discriminator. Noise was added to each real thin-slice image X_0_ to transform it into isotropic Gaussian noise (X_T_) in T steps in the forward process, with T on the order of thousands for regular diffusion models. For 3DAD, a greater amount of noise was added with a larger step size at each forward step and in the reverse diffusion direction for fast image sampling. In the reverse process, an adversarial projector comprised of a generator *G_θ_* and a discriminator *D_θ_* was used, with the generator *G_θ_* first generating the target image ${\hat{X}}_{0}$ from X_T_ and the compressed thick-slice y. Then, a denoised image sample ${\hat{X}}_{t-k}$ was synthesized from the denoising distribution q(x_t−k_|x_t_,${\hat{x}}_{0}$), and *D_θ_* discriminated the actual X_t−k_ from the synthetic sample ${\hat{X}}_{t-k}$.

We trained different models with different compression rates by compressing every two to six thin slices (compression ratio 2:1 to 6:1) to one thick slice and using thick-slice CT as the source and thin-slice CT as the ground truth. The thin-slice images were compressed by selecting the maximum of every 2 slices, up to 6 slices, to produce corresponding thick-slice images [22].

For the base dataset, the internal dataset was split randomly with a 7:3 ratio into training and validation datasets. Information from DICOM files with HU was in the range of [−160, 240] to exclude extraneous features. Augmentation enhancement strategies included rotation by angles in [−5, 5] along each of the three-dimensional coordinate axes using torchvision. Image data crop was performed with Monai (1.3.0) [23]. Each image was cropped by selecting the foreground and a random spatial by the specific size of the region of interest (ROI). To keep the same shape, the cropped images were resized into 128 × 128 × 96. After normalization to [−1, 1], the images were fed to the model for training. The time step size was set to 250, and 4 diffusion steps were used in the model. The learning rate of the generator was 1 × 10^−4^, and the discriminator was 6 × 10^−5^. The weight of the L1 loss part of the diffusion model was 0.5. Deep learning models were built with PyTorch 1.13.1 and trained on a deployed high-performance computing platform (Dell Technologies, Round Rock, TX, USA) equipped with an Intel(R) Xeon(R) Gold 6334 CPU @ 3.60 GHz (Intel, Santa Clara, CA, USA) with 32 double-threaded cores, 512 GB of memory, and 4 Nvidia A100 (80 GB) GPUs (Nvidia, Santa Clara, CA, USA).

For fine-tuning on a real-world dataset with both a ≤1.25 mm thin slice and a 5 mm thick slice, the trained parameters from the abdomen base dataset were used as the initial weights of the 3DAD model. Real-world images with ≤1.25 mm thickness (thin slices) and 5 mm thickness (thick slices) were used as the ground truth and source images, respectively. The parameter settings were kept unchanged. The dataset for fine-tuning was split into training, validation, and test sets, with two thicknesses (1 mm vs. 5 mm and 1.25 mm vs. 5 mm) used for training, 1.25 mm images for validation, and 1 mm images for testing. No non-contrast-phase cases were involved in the fine-tuning training dataset. Given the limited sample size, all three phases of CT images were used in model training (fine-tuning) to increase sample size and data diversity.

### 2.4. The Evaluation of Diagnosis of HCC

To evaluate the performance of synthetic thin-slice CT in medical applications, a published 3D convolutional network, ST3DCN, was used to diagnose HCC at the observation and patient levels based on a cropped observation region from real thin-slice and synthetic thin-slice images, using DeLong’s test to compare ROC curve similarity [24]. For internal and external abdomen base datasets with liver observations labeled, the CT images were cropped using observation masks, and the cropped images were resized to feed them to ST3DCN. The cropped images can reduce the influence of unrelated regions.

### 2.5. Radiologist Review

Two board-certified radiologists with at least thirteen years’ experience in abdominal imaging were invited to review the quality of synthetic images. The criteria for radiologist review are depicted in Table S1, which was modified from those of Khader et al. [25]. Three whole-image-level parameters, realistic image appearance, consistency between slices, and anatomic correctness, were evaluated on the fine-tuning testing dataset with a Likert scale from 1 to 4. Two observation-level parameters, observation existence and quality, were assessed with a binary score to describe the existence (exists: 1, does not exist: 0) and a Likert scale from 1 to 5 to describe the quality: 1 = poor, 2 = fair, 3 = moderate, 4 = good, 5 = excellent. These were assessed according to the following criteria: (1) Is the observation clearly visible on the image? (2) Is the boundary of the observation clearly visible in the image? (3) Can the size and shape of the observation be accurately determined based on the image? Both radiologists were blinded to the opposing assessment and to the corresponding clinical data for the collected scans.

The axial, sagittal, and coronal planes were considered, and only the largest observation was included in the quality assessment, with the size and location of the observation provided to the radiologists for reference. The size and location of the observation were determined using an automatic segmentation tool (SenseCare V2.7.6.1, SenseTime, Hong Kong, China) [26] and reviewed by experienced medical staff. The reviewed observation includes HCC, hemangioma, and cysts. To avoid recall bias, each radiologist reviewed all images twice, with real thin-slice and synthetic thin-slice images mixed in a random order, with at least 1 week as the washout period. ITK-SNAP (Version 3.9.0 and 4.2.0) was used for reviewing.

Another board-certified radiologist with 16 years’ experience in abdominal imaging was invited to diagnose the Liver Imaging Reporting and Data System (LI-RADS) as observation for each real and synthetic thin-slice CT image, with the top two observations in size considered.

### 2.6. Statistical Analysis

Continuous values were expressed in mean (±standard deviation) or median (interquartile range) as appropriate. Statistical analysis was performed using Python 3.10.14 (Python Software Foundation) and scikit-learn (1.4.0). The two-sided *p*-values < 0.05 are considered statistically significant.

To evaluate the quality of synthetic images relative to real images, MSE, PSNR, and SSIM from scikit-image (0.22.0) were used. AUCs were calculated to assess the overall diagnostic accuracy of the ST3DCN models on both original and generated images, with DeLong’s test used to compare ROC curves [27]. The 95% CI was obtained by using Hanley and McNeil’s method [24]. Pearson’s Chi-Square test was used to assess differences in radiologists’ scores between real and synthetic images.

The present study has been approved by the Institutional Review Board of the different participating institutions, in accordance with the Declaration of Helsinki.

## 3. Results

### 3.1. Dataset Characteristics

An abdomen base dataset with only real thin-slice CT from seven centers and a fine-tuning dataset with both real thin and thick slices from two centers were collected for developing and fine-tuning the 3DAD model [24]. For the training and internal validation cohort, from the 2281 patients from six centers, there were a total of 2264 patients (99.3%) included in this analysis, 1584 (976 [61.6%] male; mean [±standard deviation (SD)] age, 58.2 [±14.4]) for training and 680 (415 [61.0%] male; mean [±SD] age, 58.8 [±13.9]) for internal validation (Figure 1 and Table 1). Altogether 2485 (median [interquartile ranges (IQRs)] size, 21.3 [12.8–42.9]) and 415 (median [IQRs] size, 21.2 [12.8–39.3]) observations were denoted, with 539 (21.7%) and 249 (23.3%) categorized as HCC for training and internal validation datasets, respectively. Baseline characteristics of 548 patients (437 [79.7%] male; mean [±SD] age, 54.2 [±11.7]) and 712 observations (median [IQRs] size, 47.8 [17.5–98.3]) from the independent external cohort are depicted in Table 1, with 351 (49.3%) observations having a ground truth diagnosis of HCC.

The 133 patients (74 with 1 mm thickness and 59 with 1.25 mm thickness) for fine-tuning (Figure 1) were split into training (64 [48.1%]; 36 [56.3%] male; mean [±SD] age, 62.9 [±14.9]), validation (26 [19.5%]; 14 [53.8%] male; mean [±SD] age, 65.6 [±11.3]), and testing (43 [32.3%]; 25 [58.1%] male; mean [±SD] age, 64.4 [±13.4]), with a median observation size of the testing dataset of 20.0 and an interquartile range of 10.5 to 33.5 mm (Table 1).

### 3.2. Performance on the Base Dataset

**Figure 3:**
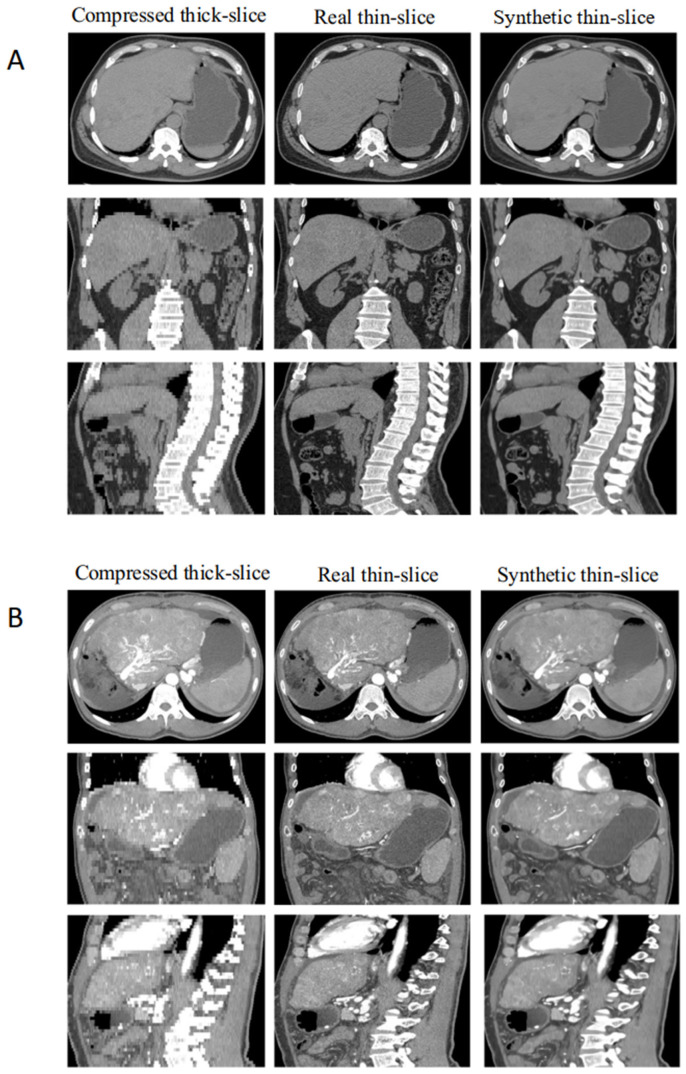

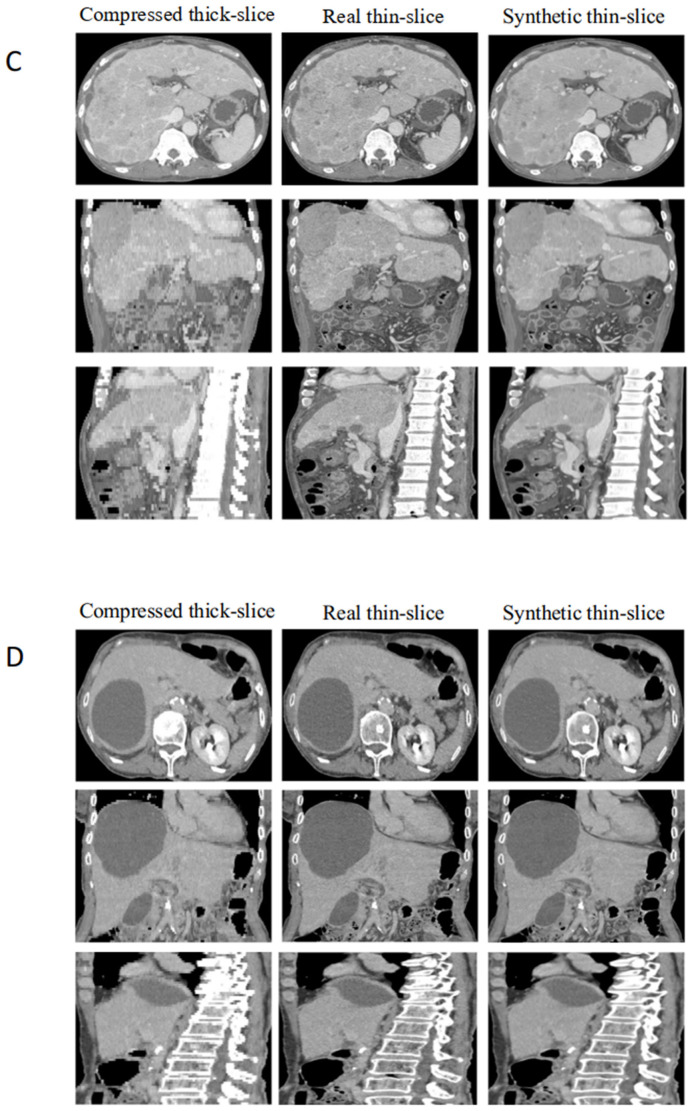
Comparison of the real thin slice and the compressed thick slice to the synthetic thin slices for different phases of the internal base dataset. The images presented were compressed and synthesized in the 5-slice scenario. (**A**) Non-contrast phase; (**B**) arterial phase; (**C**) portal-venous phase; and (**D**) delayed phase.

**Figure 4:**
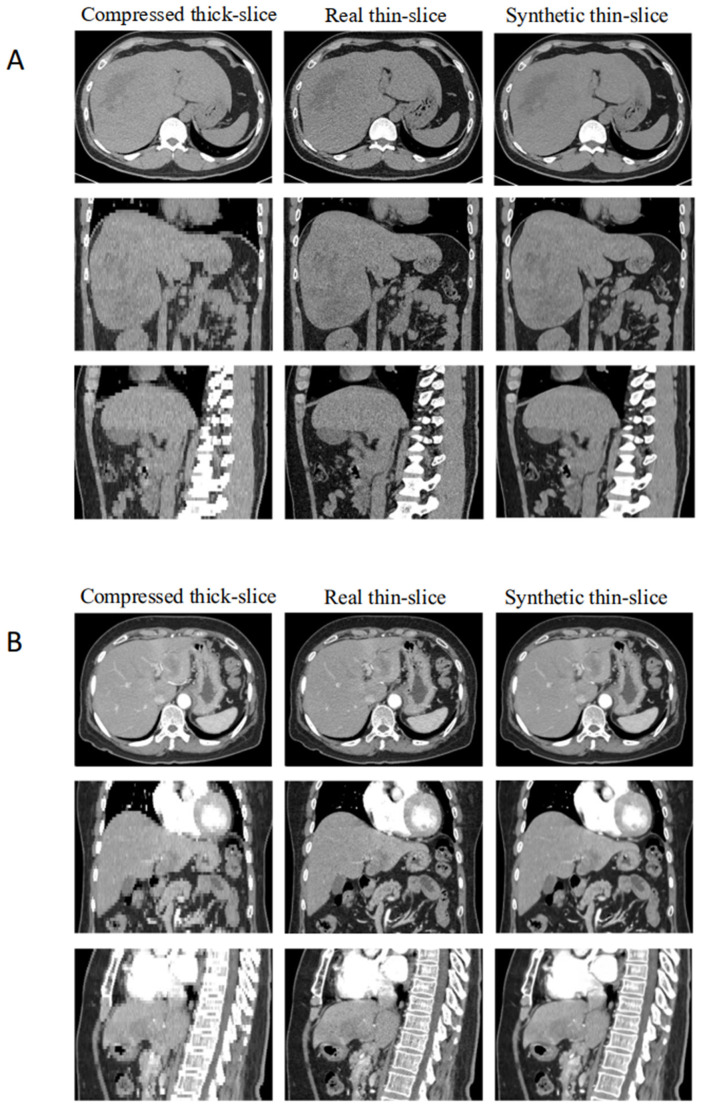

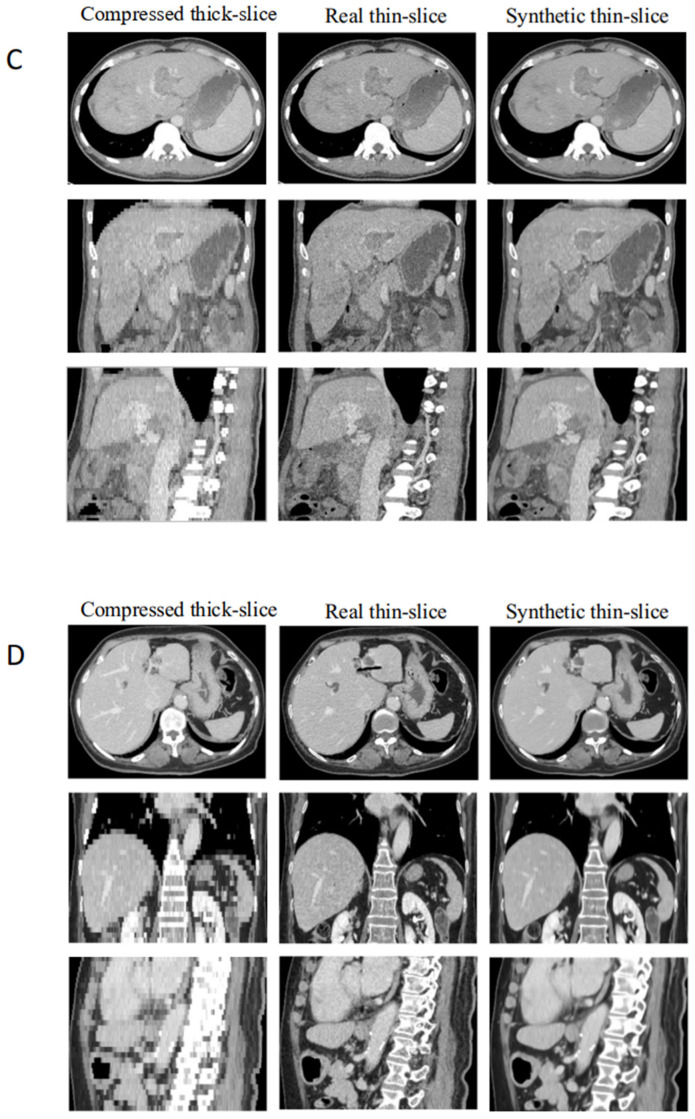
Comparison of the real thin slice and the compressed thick slice with the synthetic thin slices for different phases of the external base dataset. The images presented were compressed and synthesized in the 5-slice scenario. (**A**) Non-contrast phase; (**B**) arterial phase; (**C**) portal-venous phase; and (**D**) delayed phase.

Models from two-slice to slice-slice scenarios were trained on portal-venous-phase CT images, and evaluation was extended to non-contrast, arterial, and delayed-phase CT images. For the five-slice scenario (thin slice with a thickness ≤ 1.25 mm and compressed thick slice with a thickness of 5–6.25 mm) in the portal-venous phase, MSE, PSNR, and SSIM values of 66.346 (±37.114), 30.491 (±2.105), and 0.915 (±0.027) were achieved for internal data and 81.374 (±35.117), 29.478 (±1.935), and 0.916 (±0.027) were achieved for external data, respectively (Table 2). While extended to the arterial phases, 3DAD achieved better MSE, PSNR, and SSIM values of 62.497 (±30.940), 30.646 (±1.928), and 0.921 (±0.025) for internal data and a better SSIM value of 0.925 (±0.026) for external data. Relatively worse performance was observed in the non-contrast and delayed phases. Performance across all three metrics on external data was slightly lower than on internal data. Representative images of all four phases were displayed in Figure 3 and Figure 4 for axial, coronal, and sagittal planes. Blurred, compressed thick images (five slices to one) can be found in the sagittal and coronal planes, with the vertebrae indistinguishable. Using the compressed, blurred image as a source, 3DAD can synthesize high-resolution thin slices that closely match real thin-slice images. The performance of 3DAD dropped as the compression ratio increased from 2:1 to 6:1, across all three metrics (Table S2) for all four phases and both internal and external datasets, indicating that the quality of the synthetic image was affected by the thickness of the thick-slice CT. The performance of 3DAD, compared to Pix2pix, CycleGAN, and 2.5-DAD, is depicted in Table S3. 3DAD achieved the lowest MSE (*p* < 0.001) and highest PSNR (*p* < 0.001) and SSIM (*p* < 0.001) for both internal and external datasets from two-slice to six-slice scenarios at the portal-venous phase.

### 3.3. Performance of the Dataset for Fine-Tuning

**Figure 5:**
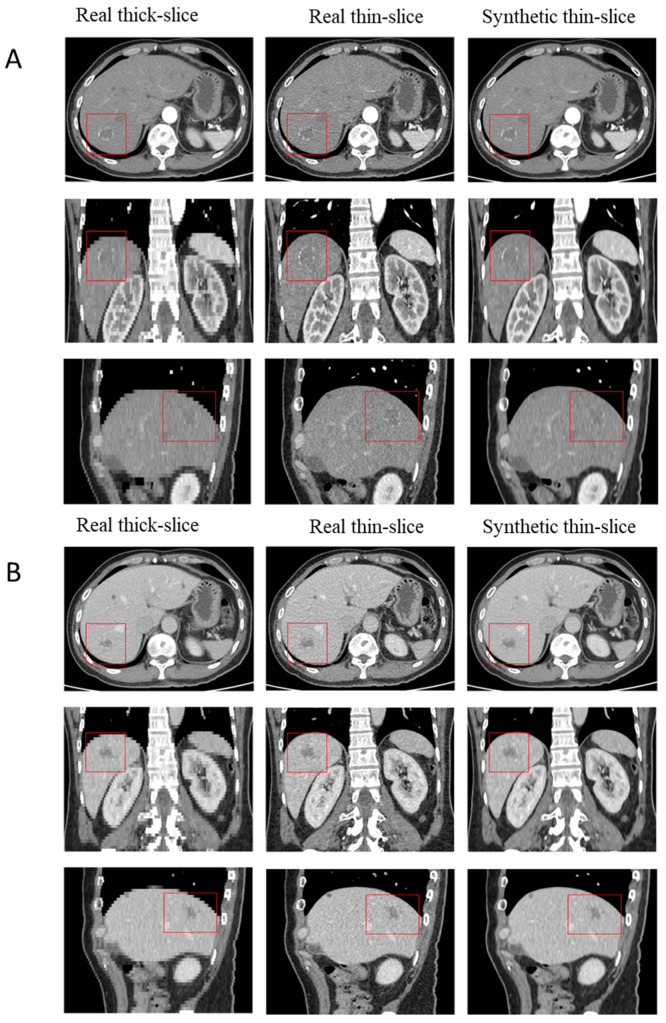
Comparison of the real thin slice and the real thick slice with the synthetic thin slices for different phases of the internal dataset after fine-tuning. The model was fine-tuned under the 5-slice scenario. (**A**) Arterial phase; (**B**) portal-venous phase.

**Figure 6:**
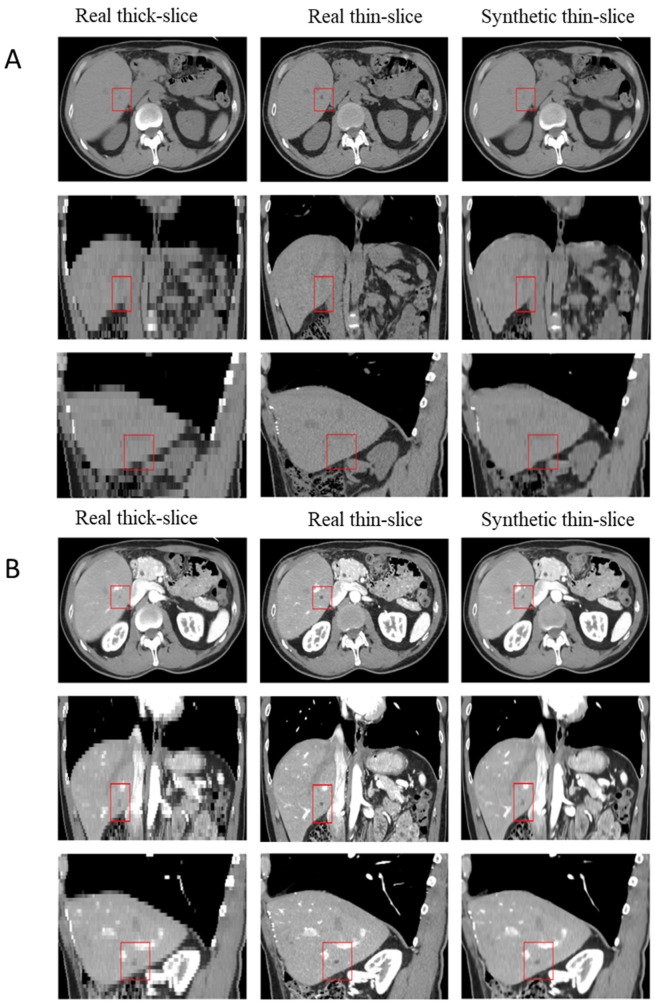

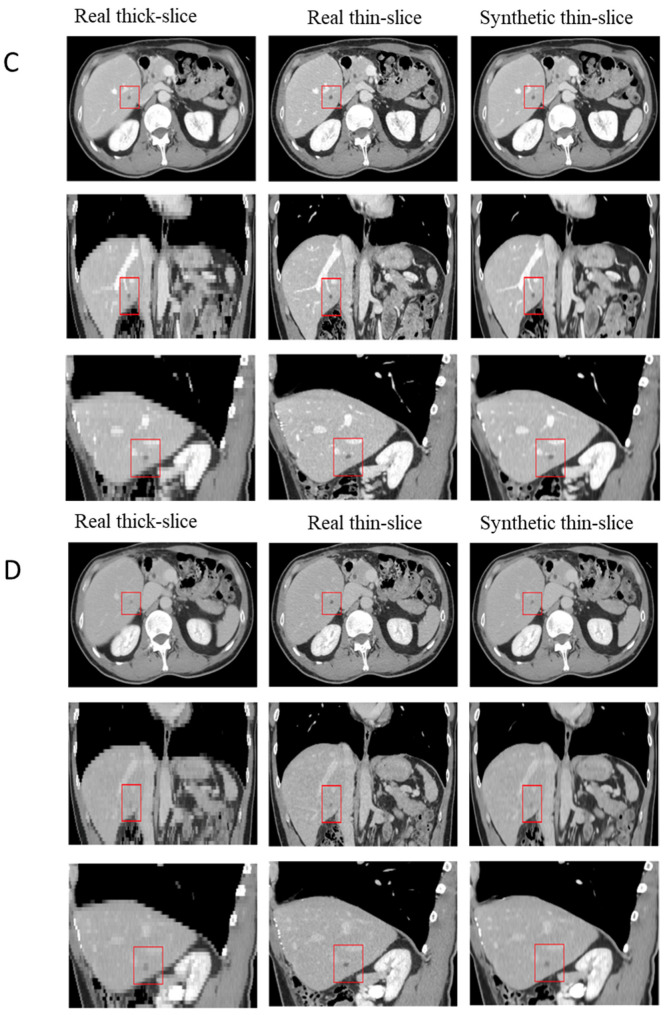
Comparison of the real thin slice and the real thick slice with the synthetic thin slices for different phases of the testing dataset after fine-tuning. The model was fine-tuned under the 5-slice scenario. (**A**) Non-contrast phase; (**B**) arterial phase; (**C**) portal-venous phase; and (**D**) delayed phase.

The evaluation of 3DAD on abdominal images was performed on the fine-tuned five-slice 3DAD model. In the 26 arterial-phase CT images in the validation dataset, MSE, PSNR, and SSIM values of 131.499 (±93.183), 27.571 (±2.061), and 0.904 (±0.030), respectively, were achieved, while the portal-venous phase obtained values of 109.534 (±53.239), 28.059 (±1.524), and 0.907 (±0.025), respectively (Table 3). For evaluating the 43 testing CT images, the performance was slightly better with MSE, PSNR, and SSIM values of 75.022 (±68.194), 29.897 (±1.607), and 0.940 (±0.023), respectively, for the arterial phase, and the portal-venous phase obtained values of 70.435 (±70.233), 30.243 (±1.710), and 0.940 (±0.023), respectively. The best performance on the fine-tuning data was in the delayed phase of the testing dataset, with MSE, PSNR, and SSIM values of 54.992 (±11.332), 30.817 (±0.897), and 0.943 (±0.016), respectively. When evaluating directly on non-contrast-phase images without non-contrast-phase images for fine-tuning, performance decreased slightly, with MSE, PSNR, and SSIM values of 194.501 (±32.885), 25.301 (±0.740), and 0.876 (±0.019), respectively. Representative images are displayed in Figure 5 and Figure 6. 3DAD can generate high-fidelity thin-slice CT images with low noise and artifacts from real-world thick-slice CT images.

### 3.4. Diagnosis of HCC

**Figure 7:**
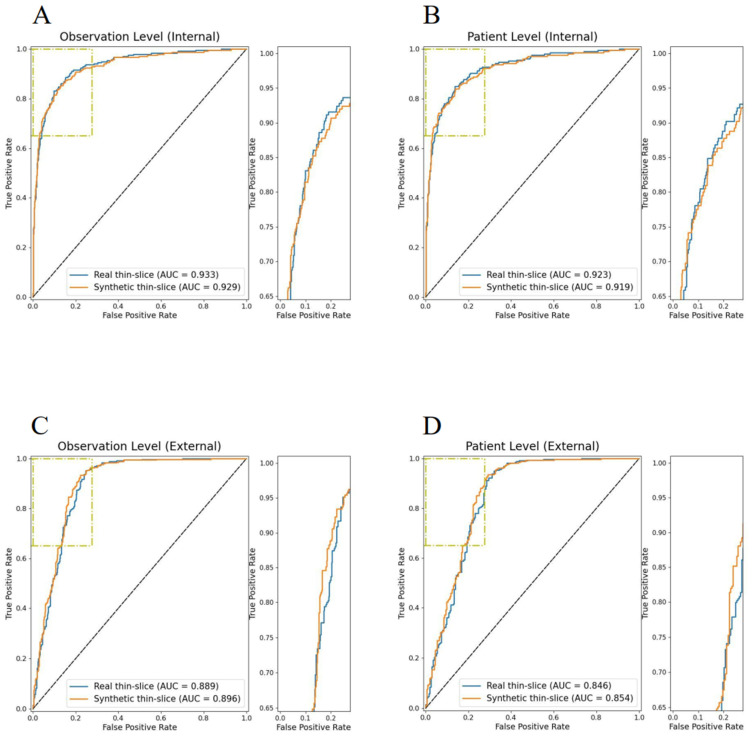
AUC of real thin slice compared to synthetic thin slice in the diagnosis of HCC for 5-slice scenarios at the observation and patient level. (**A**) Observation level for internal dataset; (**B**) patient level for the internal dataset; (**C**) observation level for the external dataset; (**D**) patient level for the external dataset.

The ROC curve was used to show the performance of HCC diagnosis compared to the synthetic thin-slice images to the real thin-slice images (Figure 7). Synthetic thin-slice images achieved slightly lower AUCs at both the observation and patient levels for internal data and slightly higher AUCs at both levels for external data across all compression ratios (Table S4). The best two-slice scenario achieved an AUC of 0.93 for the synthetic thin-slice image versus 0.933 for the real thin-slice image at the observation level and 0.92 for the synthetic thin-slice image versus 0.923 for the real thin-slice image at the patient level on internal data. For external data, the worst two-slice scenario achieved AUCs of 0.891 for the synthetic thin-slice image versus 0.889 for the real thin-slice image at the observation level and 0.848 for the synthetic thin-slice image versus 0.846 for the real thin-slice image at the patient level. DeLong’s test showed no significant difference in paired ROC comparisons for the internal dataset (*p* ≥ 0.062). In contrast, a significant difference was found int he four-, five-, and six-slice scenarios at the observation level (*p* ≤ 0.029) and the four- and six-slice scenarios at the patient level (*p* ≤ 0.035) for the external dataset (Table S4). The non-significant difference in the internal dataset and partial significance in the external dataset indicated 3DAD’s capability in generating trustable thin slices for downstream analysis.

### 3.5. Radiomics Features Analysis

The differences in reproducibility of radiomics features across different image types are depicted in Table S5 (synthetic thin slice vs. real thin slice) and Table S6 (real thick slice vs. real thin slice) [6,14]. For the 17 types, including total, original image type, and all the other filtered types, all CCCs for synthetic thin slice vs. real thin slice significantly increased compared to the CCCs for real thick slice vs. real thin slice for the arterial phase, portal-venous phase, and delayed phase (*p* < 0.048), while nine showed a significant increase for the non-contrast phase (*p* < 0.026). The heatmap of CCCs is depicted in Figure S1. In all four phases, the increased presence of green and reduced magenta indicates that the synthesis from real thick images to synthetic thin images yields higher CCC values than synthesis from real thin images.

### 3.6. Radiologists Review

**Figure 8:**
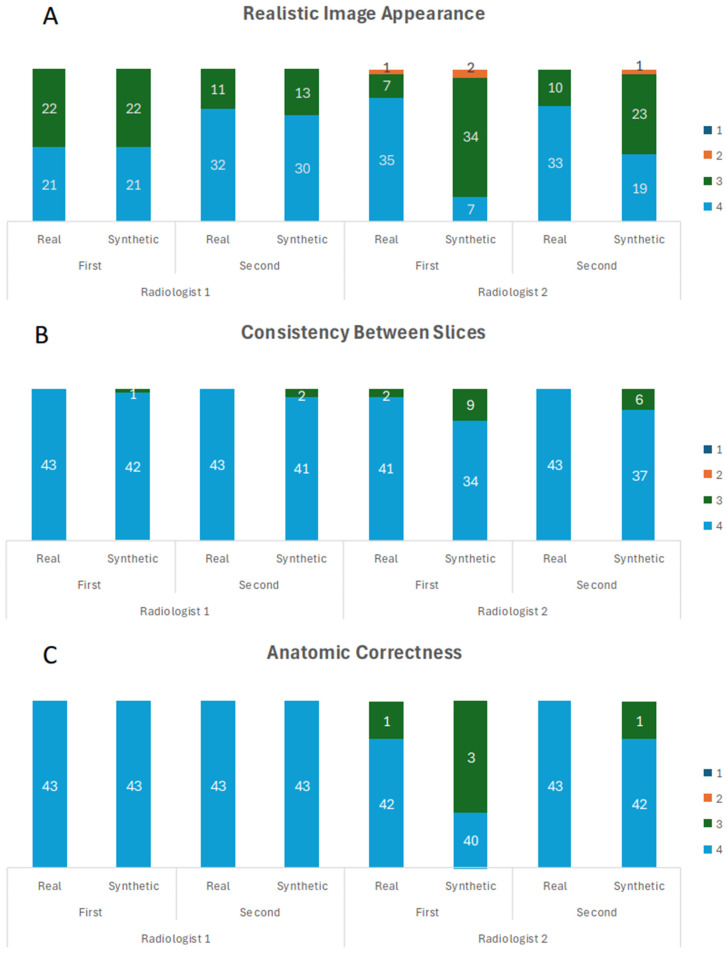

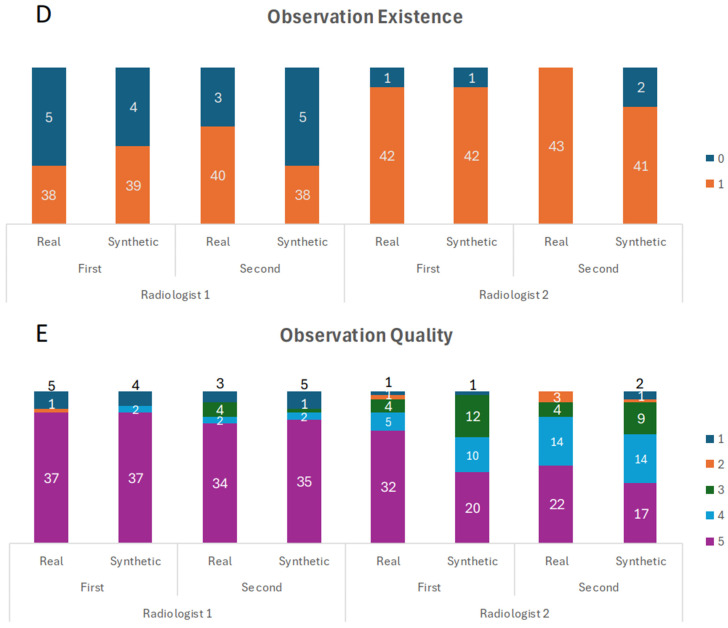
Quantitative evaluation by radiologists by comparing the real and synthetic thin slices. Two radiologists reviewed the images twice after mixing real and synthetic images in random order, and the image order in the first review was different from that in the second. (**A**) Realistic image appearance; (**B**) consistency between slices; (**C**) anatomic correctness; (**D**) observation existence; (**E**) observation quality.

The quantitative evaluation results by the two radiologists are presented in Figure 8. Radiologist 1 rated all 172 (43 × 4) images as overall realistic with only minor unrealistic areas or above (43/43 for real for both the first and second review, 43/43 for synthetic for both the first and second review), while radiologist 2 rated 168 of 172 images correspondingly (42/43 for real of the first review, 41/43 for synthetic of the first review, 43/43 for real of the second review, and 42/43 for synthetic of the second review) (Figure 8A). A total of 169 of 172 images were consistent among all slices by radiologist 1 (43/43 for the real of the first and second review, 42/43 for the synthetic of the first review, and 41/43 for the second), while radiologist 2 considered 155 of 172 correspondingly (41/43 for real of the first review, 34/43 for synthetic of the first review, 43/43 for real of the second review, and 37/43 for synthetic of the second review) (Figure 8B). All images were evaluated as consistent between most slices for both radiologists in the two reviews.

All images were assessed correctly for anatomical features for both the first and the second review and both real and synthetic images by radiologist 1. In contrast, five of 172 images were assessed as minor anatomic incorrectness and 167 of 172 as correct (Figure 8C). Radiologist 1 did not find 17 of 172 observations (5/43 for the real of the first review, 4/43 for the synthetic of the first review, 3/43 for the real of the second review, and 5/43 for the synthetic of the second review), while four of 172 observations were missed by radiologist 2 (1/43 for both real and synthetic for the first review and 2/43 for synthetic of the second review) (Figure 8D).

One hundred and forty-nine of 172 observations were assessed as good or excellent by radiologist 1 (37/43 for real of first review, 39/43 for synthetic of first review, 36/43 for real of second review, and 37/43 for synthetic of second review). In comparison, 134 of 172 observations were assessed as good and excellent by radiologist 2 (37/43 for real of first review, 30/43 for synthetic of first review, 36/43 for real of second review, and 31/43 for synthetic of second review) (Figure 8E). The synthetic images were assessed as realistic and high-quality by the radiologists. The observations that could not be found by the radiologists (observation existence: 0 in Figure 8D) were assigned a score of 1 for observation quality assessment (Figure 8E).

To evaluate the difference between real and synthetic images in each review, Pearson’s Chi-Square test was calculated (Table 4). By comparing the review scores between the real and synthetic images in the first and second reviews by radiologist 1, no significant difference was found. Significant differences were found for the parameters realistic image appearance and consistency between slices by radiologist 2, which was caused by the difference in scores between grade 3 (overall realistic and only minor unrealistic areas) and grade 4 (cannot tell whether fake or not) for realistic (Figure 8A) and grade 3 (majority of slices are consistent) and grade 4 (all slices are consistent) for consistency (Figure 8B).

Fifty observations in 51 were diagnosed with the same LI-RADS category for real and synthetic thin-slice CT images, indicating that the synthetic thin-slice CT images show success in liver LI-RAD diagnosis and have potential in other medical applications (Figure S2).

## 4. Discussion

The present study proposed a novel 3D diffusion model, 3DAD, by applying a larger diffusion step to significantly shorten training time and enabled training the 3D diffusion model for CT image generation. It is the first three-dimensional adversarial diffusion model for translating compressed ≥ 5 mm-thick-slice CT to ≤1.25 mm-thin-slice CT with excellent performance and systematic evaluation. Specific models were trained from two to six slices, with MSE, PSNR, and SSIM achieving 62.497–68.650, 28.533–30.740, and 0.904–0.925, respectively, across all four phases in the five-slice scenario for both internal and external datasets. While evaluated on real-world data with both ≤1.25 mm thin-slice CT and 5 mm thick-slice CT in the five-slice scenario after fine-tuning on a small dataset, the achieved performance of MSE, PSNR, and SSIM was 109.534, 28.059, and 0.907 for validation and 70.435, 30.243, and 0.940 for testing, respectively, in the portal-venous phase. The high performance indicates 3DAD’s capability on simulated and real-world CT images, which may facilitate the reuse of the large compressed 5 mm CT images in clinical applications and medical research, especially in big-data-driven AI times.

The performance of 3DAD was robust, demonstrating strong performance across different clinical and non-clinical scenarios. Previous studies lack a comprehensive approach due to difficulties in obtaining large, well-curated cohorts. Kudo et al. [14] focused on the methodology for the head, chest, abdomen, and legs with fewer than 100 CT images obtained for each part, and no downstream clinic testing or radiologist evaluation was performed. The present study obtained 2264 abdominal CTs from Asian patients and generalized the model to an external dataset, achieving high performance across all three evaluation metrics (Table 2). The acquisition of CT volumetric data collected from multiple institutes with diverse scanners and image acquisition protocols was used for scanning [24], with images obtained from all four major global CT manufacturers (Siemens, GE, Philips, and Toshiba), indicating that the model can handle varying acquisition protocols, scanner variability, and patient demographics. The 3DAD model was trained only on portal venous phase CT images but performed well on non-contrast-, arterial-, and delayed-phase CT images (Table 2), indicating that 3DAD is generalizable across phases, avoiding the need to train separate models for each phase or to train a model on images from all phases.

Furthermore, after fine-tuning on a small dataset, 3DAD achieved even higher performance compared to the base dataset for the five-slice model. It should be noted that only 13 delayed-phase CT images were used for fine-tuning, yet the achieved performance was the best for the delayed phase compared to the other three phases on the testing dataset (Table 3). No non-contrast-phase images were involved in fine-tuning training, which may explain its lower performance compared to other phases (Table 3). Because CT machine settings vary, the thickness of CT images from different hospitals or medical centers may differ. Combining CT images of different thicknesses in the training of a single model, rather than training separate models for each thickness, is necessary. After assessing thicknesses of 1 mm and 1.25 mm, we achieved MSE, PSNR, and SSIM values of 53.497, 31.048, and 0.928, respectively, for 1 mm and 87.459, 29.007, and 0.904 for 1.25 mm images because of different thicknesses (5 mm vs. 6.25 mm in thick slice for 1 mm vs. 1.25 mm in thin slice) in the five-slice scenario in the portal-venous phase for the test set. After fine-tuning, the performance of MSE, PSNR, and SSIM was 109.534, 28.059, and 0.907 for validation and 70.435, 30.243, and 0.940 for testing, respectively. The distribution shifted across different thicknesses, but their influence was eliminated or reduced after fine-tuning. However, more research is still needed to understand how performance is influenced by combining different thickness images in a single model.

The radiologists’ evaluations approved the high quality of the synthetic thin-slice CT at the whole-image and observation levels. From the first and second reviews of both radiologists, for both real and synthetic images, high-quality metrics were achieved for image realism, consistency, and anatomical correctness (Figure 8). Although significant differences were observed between real and synthetic thin-slice CT in terms of realism and consistency, these differences arose from scores indicating that radiologists could distinguish the images while still rating the synthetic ones as high-quality. Misses in observations occurred for both real and synthetic images (Figure 8E), suggesting they stemmed from the review process rather than synthesis flaws and were likely influenced by the small size of the observations (median 0.6 cm). Thus, these shortcomings did not undermine the study results, as they demonstrated comparable visibility between synthetic and real images.

The present study has several limitations. First, different models were trained for two-slice to six-slice scenarios, thereby increasing the number of trained models and the training resources. This can be resolved by the study of Kudo et al. [14], which proposed a conditional discriminator to handle different body parts and slice thicknesses. Second, although a larger step size in 3DAD replaced the small step size in the regular diffusion model, approximately 500 h was required to train the abdomen base dataset on a single GPU with 80 GB of memory. 3DAD enabled training a three-dimensional diffusion model for CT image synthesis but still required a notably longer training time than Pix2pix and CycleGAN. Further improvements in computational efficiency are still needed. Third, although the performance of the present model in HCC diagnosis and radiologist evaluation is impressive, it was not systematically assessed for detecting small lesions (≤5 mm). Comparisons between synthetic images and real thin-slice CT for challenging clinical tasks, such as detecting microvascular invasion, assessing tumor capsule integrity, and identifying small-vessel tumor thrombi, were not conducted.

## 5. Conclusions

In conclusion, the novel 3DAD, a three-dimensional adversarial diffusion model, showed excellent performance in synthesizing high-resolution thin-slice CT images from thick-slice images. 3DAD applied a fast diffusion process for efficient training and leveraged a conditional adversarial generator to handle different slice thicknesses. The quality of synthetic thin-slice CT was confirmed by generalization across different CT phases, application to HCC diagnosis, and radiologist assessment. If properly applied, 3DAD can promote the reuse of large, thick-slice CT images in medical systems and facilitate high-fidelity volume images in clinical diagnosis and medical research.

## Figures and Tables

**Table 1:** Baseline characteristics of patients undergoing model training and fine-tuning.

		All Patients	Interquartile Range
Base			
Training	Age (years)	58.2 (±14.4)	48–68
	Male patients (%)	976 (61.6%)	
	Observation number:	2485	
	HCC	539	
	Non-HCC	1946	
	Observation size (mm)	21.3 ^1^	12.8–42.9
Internal validation	Age (years)	58.8 (±13.9)	50–68
	Male patients (%)	415 (61.0%)	
	Observation number:	1070	
	HCC	249	
	Non-HCC	821	
	Observation size (mm)	21.2	12.8–39.3
External validation	Age (years)	54.2 (±11.7)	46–63
	Male patients (%)	437 (79.7%)	
	Observation number:	712	
	HCC	351	
	Non-HCC	361	
	Observation size (mm)	47.8	17.5–98.3
Fine-tune			
Training	Age (years)	62.9 (±14.9)	58–73
	Male patients (%)	36 (56.3%)	
Validation	Age (years)	65.6 (±11.3)	59.5–72.3
	Male patients (%)	14 (53.8%)	
Testing	Age (years)	64.4 (±13.4)	53–74
	Male patients (%)	25 (58.1%)	10.5–33.5
	Observation size (mm)	20.0 ^1^	

^1^ Median used.

**Table 2:** Performance of 3DAD for the abdomen base dataset in the 5-slice scenario.

	Internal			External		
	MSE	PSNR	SSIM	MSE	PSNR	SSIM
Non-contrast	68.650 (±77.029) ^1^	30.740 (±2.475)	0.908 (±0.035)	86.493 (±45.699)	29.176 (±1.598)	0.914 (±0.026)
Arterial	62.497 (±30.940)	30.646 (±1.928)	0.921 (±0.025)	87.642 (±38.638)	29.158 (±1.912)	0.925 (±0.026)
Portal-venous	66.346 (±37.114)	30.491 (±2.105)	0.915 (±0.027)	81.374 (±35.117)	29.478 (±1.935)	0.916 (±0.027)
Delayed	66.574 (±46.252)	30.530 (±2.184)	0.913 (±0.028)	99.984 (±39.993)	28.533 (±1.820)	0.904 (±0.023)

^1^ mean (±standard deviation).

**Table 3:** Performance of 3DAD on the validation and testing dataset after fine-tuning.

	Validation			Testing		
	MSE	PSNR	SSIM	MSE	PSNR	SSIM
Non-contrast	-	-	-	194.501 (±32.885)	25.301 (±0.740)	0.876 (±0.019)
Arterial	131.499 (±93.183)	27.571 (±2.061)	0.904 (±0.030)	75.022 (±68.194)	29.897 (±1.607)	0.940 (±0.023)
Portal-venous	109.534 (±53.239)	28.059 (±1.524)	0.907 (±0.025)	70.435 (±70.233)	30.243 (±1.710)	0.940 (±0.023)
Delayed	-	-	-	54.992 (±11.332)	30.817 (±0.897)	0.943 (±0.016)

**Table 4:** Pearson’s Chi-Square test between real and synthetic thin slice for the scores from radiologists.

	Radiologist 1 ^1^	Radiologist 1 ^2^	Radiologist 2 ^1^	Radiologist 2 ^2^
Realistic image appearance	1.0	0.81	<0.001 #	0.007
Consistency between slices	1.0	0.474	0.05	0.034
Anatomic correctness	1.0	1.0	0.5	1.0
Observation existence	1.0	0.71	1.0	0.474
Observation quality	0.375	0.509	0.051	0.234

^1^ Represents the first review; ^2^ the second review. # significant when *p* < 0.05.

## Data Availability

The codes are available at https://github.com/HKUMedicineLiverAI/3DAD (access date 21 May 2026). Model weights and data generated or analyzed during the study are available from the corresponding author upon request.

## Supplementary Materials

The following supporting information can be downloaded at https://www.mdpi.com/article/10.3390/bioengineering13060595/s1, Figure S1: CCCs heatmap for 1488 radiomics features for all image types; Figure S2: LIRADS diagnosis between real and synthetic thin-slice CT images; Table S1: Overview of the categories for the three whole-image level parameters for radiologist review; Table S2: Performance of 3DAD at different phases of the abdomen base dataset; Table S3: Performance of benchmarks at the portal-venous phase of the abdomen base dataset; Table S4: AUCs for the diagnosis of HCC were compared between synthetic and real thin-slice CT for the abdomen base dataset using ST3DCN; Table S5: CCC per image filter types between real and synthetic thin-slice CT for fine-tuning testing data; Table S6: CCC per image filter types between real thin-slice and real thick-slice (5 mm) CT images for fine-tuning testing data; References [13,22,24,26,28,29] are cited in the Supplementary Materials.
