# Supplementary material for: 3DAD: Super-Resolution Image Synthesis from Anisotropic CT Images Using a Three-Dimensional Adversarial Diffusion Model
Source: Bioengineering (Basel). 2026 May 22;13(6):595. doi: 10.3390/bioengineering13060595 (PMC13296151; doi:10.3390/bioengineering13060595)
Supplement: Supplementary file 1 [file bioengineering-13-00595-s001.zip › bioengineering-4263632-supplementary.pdf]

3DAD: Super-Resolution Image Synthesis from Anisotropic CT Images Using a Three-Dimensional Adversarial Diffusion Model

Jianliang Lu, Ho Ming Cheng, Benjamin Xin Hao Fang, Chun On Anderson Tsang, Sarah Yu, Wai-Kay Seto, Philip Leung Ho Yu & Keith Wan-Hang Chiu

Supplementary Appendix

Table of Contents

Section S1: Methods **3**
Image pre-processing 3
Basic denoising diffusion model 3
3DAD model 4
Radiomics feature analysis 6
Section S2: Supplementary Tables **8**
Section S3: Supplementary Figures **16**
Reference **18**

Section S1: Methods

Image pre-processing

All CT scans were in DICOM format. The acquisition of computed tomography volumetric data collected from multiple institutes with diverse scanners and image acquisition protocols was used for scanning [1], with images obtained from all four major global CT manufacturers (Siemens, GE, Philips, and Toshiba). Collected scans underwent deidentification and removal of personally sensitive data before being uploaded to a secure cloud server (Microsoft Azure), encrypted in transit using the Internal Protocol Security/Internet Key Exchange (IPsec/IKE) via a site-to-site virtual private network.

For abdomen base datasets with liver observations, whether involved or segmented, the size of liver DICOM observations was determined by their longest measurable diameter, with a maximum of 3 observations per scan. Each observation was manually contoured and segmented by eight trained operators using ITK-SNAP 3.8, a medical imaging processing tool (itksnap.org) [2]. Each operator attended a five-hour supervised contour training session conducted by a specialist abdominal radiologist in recognizing liver observations on CT, followed by a test session involving 28 scans. All operators were required to achieve a dice similarity coefficient of >0.6 (i.e., substantial agreement) with the radiologist before proceeding to actual contouring [1]. We contoured all observations on arterial and porto-venous phases, with additional contouring of the late phase arranged for indistinct observations (e.g., isodense or not visible on porto-venous phase imaging) after specialist radiological review.

Further image optimization was required to facilitate standardized processing. With differences in image intensities, we windowed the range of Hounsfield units HU to [-160, 240]. We normalized the image to [-1, 1] to remove extraneous features, ensuring the optimal settings for subsequent deep learning [1]. The raw image size of CT scans was 512 (height in pixels) ×512 (width in pixels) ×N, where N denoted the number of slices.

Basic denoising diffusion model

The development of the three-dimensional model for image reconstruction was based on the basic denoising diffusion model. This model included a forward and backward process that mapped between real images and pure noise samples over a gradual T-time-step process. In the forward process, a small amount of Gaussian noise was added to the original image $\boldsymbol{x}_0$ at each step, and after sufficient T steps, the original image was converted into a noise sample $\boldsymbol{x}_t$ from an isotropic Gaussian distribution. The forward process of diffusion was a Markov chain with respective forward transition probability from $\boldsymbol{x}_{t-1}$ to $\boldsymbol{x}_t$:

$$\boldsymbol{x}_t = \sqrt{1-\beta_t}\boldsymbol{x}_{t-1} + \sqrt{\beta_t}\boldsymbol{\varepsilon}, \tag{1}$$

$$q(\boldsymbol{x}_t|\boldsymbol{x}_{t-1}) = \mathcal{N}(\boldsymbol{x}_t; \sqrt{1-\beta_t}\boldsymbol{x}_{t-1}, \beta_t\boldsymbol{I}), \tag{2}$$

where $\boldsymbol{\varepsilon}$ is added noise, β_t is noise variance, $\mathcal{N}$ represents a Gaussian distribution, and $\boldsymbol{I}$ represents an identity covariance matrix. Similarly, the reverse diffusion process was also a

Markov chain gradually denoising from $\boldsymbol{x}_t$ to $\boldsymbol{x}_0$. The reverse transition probability represented by a Gaussian distribution from $\boldsymbol{x}_t$ to $\boldsymbol{x}_{t-1}$ is:

$$p_\theta(\boldsymbol{x}_{t-1}|\boldsymbol{x}_t) = \mathcal{N}(\boldsymbol{x}_{t-1};\ \boldsymbol{\mu}(\boldsymbol{x}_t, t), \textstyle\sum(x_t, t))\,. \quad (3)$$

The diffusion model was trained to calculate $p(\boldsymbol{x}_0)$ to represent the true data distribution $q(\boldsymbol{x}_0)$ through each reverse diffusion step by estimating $\boldsymbol{\mu}$ and $\sum$ using a neural network. The following variational bound on log-likelihood was needed to minimize the training:

$$L_{vb} = \mathrm{E}_q\left[log\frac{p_\theta\,(x_{0:T})}{q(x_{1:T}|x_0)}\right] \leq \mathrm{E}_{q(x_0)}[\log p_\theta(x_0)], \quad (4)$$

where E_q is expectation over q, q denotes the network parameters. $x_{0:T}$ is the collection of image samples between time steps 0 and T, and $x_{1:T}\,|\,x_0$ represents a collection of image samples between time steps 1 to T under the condition of the sample at time step 0. Equation 4 can be decomposed into:

$$L_{vb} = log\,p_\theta(x_0\,|\,x_1) - \sum_{t=1}^{T} KL(q(\boldsymbol{x}_{t-1}|\boldsymbol{x}_t, x_0)\,\|p_\theta\,(x_{t-1}\,|\,x_t))\,, \quad (5)$$

where KL is the Kullback-Leibler divergence. After defining $\alpha_t = 1 - \beta_t$ and $\overline{\alpha_t} = \prod_{s=0}^{t} \alpha_s$, we can omit $\sum$ but focus on $\boldsymbol{\mu}$:

$$\boldsymbol{\mu_\theta}(x_t, t) = \frac{\mathbf{1}}{\sqrt{\boldsymbol{\alpha_t}}}\left(\boldsymbol{x_t} - \frac{\boldsymbol{\beta_t}}{\sqrt{\mathbf{1} - \overline{\boldsymbol{\alpha_t}}}}\boldsymbol{\epsilon_\theta}(x_t, t)\right). \quad (6)$$

Instead of parameterizing $\boldsymbol{\mu_\theta}(x_t, t)$ as a neural network directly, we can train a model $\boldsymbol{\epsilon_\theta}(x_t, t)$ to predict the added noise ϵ using the following loss function [3]:

$$L_{loss} = \mathrm{E}_{t,x_0,\epsilon}\left[\left\|\epsilon - \epsilon_\theta\left(\sqrt{\overline{\alpha_t}}\,x_0 + \sqrt{1 - \overline{\alpha_t}}\,\epsilon, t\right)\right\|_2^2\right], \quad (7)$$

where $t \in \mu(0, T), x_0\,\epsilon\,q(x_0)$ and $\epsilon \in \mathcal{N}(\boldsymbol{0}, \boldsymbol{I})$, respectively. The reverse process starts from a random sample. For each step t, $\boldsymbol{m}$ is derived by equation 6 and $\boldsymbol{x}_{t-1}$ is sampled with equation (3).

3DAD model

A novel three-dimensional adversarial diffusion (3DAD) model was developed for high-fidelity reconstruction from thick-slice to thin-slice images of a given anatomy in any body part. The diffusion model in 3DAD was equipped with a source-conditional projector that used thick-slice images to guide the reconstruction of thin-slice images during reverse sampling. 3DAD comprised a generator and a discriminator for fast, accurate mapping during reverse diffusion steps. The diffusion generator synthesizes a denoised image sample for the target thin image, given a random noise term and a source thick image as conditioning. The diffusion discriminator distinguishes between noisy samples from the actual target thin slice image and the denoised synthetic target thin slice image. A greater amount of isotropic Gaussian noise

was used with a larger step size in each forward step and the reverse diffusion direction was used for fast image sampling. The architecture of 3DAD is shown in **Figure 2.1**.

In the adversarial diffusion process, unlike the regular diffusion model, which used a large T to keep a small enough step size and satisfy the normality assumption in equation (3), a much larger step size was adopted to achieve faster diffusion in the forward and reverse processes:

$$\boldsymbol{x}_t = \sqrt{1-\gamma_t}\boldsymbol{x}_{t-k} + \sqrt{\gamma_t}\boldsymbol{\varepsilon} \tag{8}$$

$$q(\boldsymbol{x}_t|\boldsymbol{x}_{t-k}) = \mathcal{N}(\boldsymbol{x}_t; \sqrt{1-\gamma_t}\boldsymbol{x}_{t-k}, \gamma_t\boldsymbol{I}) \tag{9}$$

where k is the step size and k >> 1. The noise variance γ_t is defined as below:

$$\gamma_t = 1 - e^{\overline{\beta}_{min}\frac{k}{T} - (\overline{\beta}_{max} - \overline{\beta}_{min})\frac{2tk - k^2}{2T^2}} \tag{10}$$

where $\overline{\beta}_{max}$ and $\overline{\beta}_{min}$ were the boundary of noise variance [3].

Given k>>1, $q(\boldsymbol{x}_{t-k}|\boldsymbol{x}_t, \boldsymbol{y})$ cannot be expressed by equation (3). According to [3]:

$$q(\boldsymbol{x}_{t-k}|\boldsymbol{x}_t, \boldsymbol{x_0}, \boldsymbol{y}) = q(\boldsymbol{x}_{t-k}|\boldsymbol{x}_t, \boldsymbol{x_0}) \tag{11}$$

Based on Bayes's rule, there is:

$$q(\boldsymbol{x}_{t-k}|\boldsymbol{x}_t, \boldsymbol{x_0}) = q(\boldsymbol{x}_t|\boldsymbol{x}_{t-k}, \boldsymbol{x_0})\frac{q(\boldsymbol{x}_{t-k}|\boldsymbol{x_0})}{q(\boldsymbol{x}_t|\boldsymbol{x_0})} \tag{12}$$

Setting below parameters:

$$\overline{\boldsymbol{\mu}} = \frac{\sqrt{\overline{\alpha_{t-k}}}\gamma_t}{1-\overline{\alpha_t}}\boldsymbol{x_0} + \frac{\sqrt{\alpha_t}\,(1-\overline{a}_{t-k})}{1-\overline{\alpha_t}}\boldsymbol{x_t} \tag{13}$$

and

$$\overline{\gamma} = \frac{(1-\overline{a}_{t-k})}{1-\overline{\alpha_t}}\gamma_t \tag{14}$$

where $\alpha_t = 1-\gamma_t$ and $\overline{\alpha_t} = \prod_{r=0}^{r=t}\alpha_r$,

The equation below can be obtained from equation (8).

$$q(\boldsymbol{x}_{t-k}|\boldsymbol{x}_t, \boldsymbol{x}_0) = \mathcal{N}(\boldsymbol{x}_{t-k}; \overline{\boldsymbol{\mu}}(\boldsymbol{x}_t, \boldsymbol{x}_0), \overline{\gamma}\boldsymbol{I}) \tag{15}$$

For sampling from the network parameterized denoising distribution, a trivial albeit deterministic sample from the output of the generator is obtained:

$$p_\theta(x_{t-k}|x_t, y) \approx q\big(x_{t-k}\big|x_t, \tilde{x}_0 = G_\theta(x_t, y, t)\big) \tag{16}$$

where G_θ is the generator. $\tilde{x}_0$ is predicted at *t/k* steps away from x_t.

Given a source thick slice image y, 3DAD can generate a target thin slice image x. A pair of generator and discriminator is used with parameters G_q and D_q. The reverse process starts

from a Gaussian noise image X_T and a source thick image y. Target images are generated by the generator at each *T/k* step. The denoised target images are estimated with:

$$\hat{x}_0 = G_\theta(x_t, y, t) \quad (17)$$

Then, to sample from the denoising distribution with equation (16), there is:

$$\hat{x}_{t-k} \approx q(x_{t-k}|x_t, \hat{x}_0) \quad (18)$$

Starting from pure noise, the generator G_q removes noise gradually in each reverse step to generate $\hat{x}_{t-k} \sim p_\theta(x_{t-k}|x_t, y)$ under the source image y and x_t. The discriminator D_q distinguishes samples between true and estimated denoising distributions $q(x_{t-k}|x_t, y)$ vs. $p_\theta(x_{t-k}|x_t, y)$ with (x_t, x_{t-k}) and $(x_t, \hat{x}_{t-k})$ provided.

In the training process, the loss function of generator G_q is set as:

$$L(G) = \arg min_G max_D \, \mathrm{E}_{x,y}\left[\log\left(1 - D\left(p_\theta(x_t, y)\right)\right)\right] + \lambda E_{x,y,z}[\|x - G(y,z)\|_1] \quad (19)$$

where z represents random noise, l is the weight of the L1 loss.

The loss function of discriminator D_q is set as:

$$\arg min_G max_D \, L(D) = E_{y,x}[\log(D(q(x_t, y)))] + E_{y,z}\left[\log\left(1 - D(p_\theta(x_t, y))\right)\right] \quad (20)$$

Radiomics feature analysis

A deep learning-based automatic segmentation tool (SenseCare V2.7.6.1, SenseTime) was used for liver observation segmentation [4], performed on the original CT images of the test set for fine-tuning across all non-contrast, arterial, portal-venous, and delayed phases. Segmented observations included both high- and low-risk cases; the largest observation was identified by an experienced medical staff member, and its mask was used for subsequent radiomics feature analysis. A total of 1,488 three-dimensional radiomics features were extracted by Pyradiomics (V3.1.0) [5] from original thin scans, reconstructed thin scans, and original thick scans of the testing dataset for fine-tuning. The 1,488 radiomics features included features from unfiltered original images and 15 filtered image types using the Laplacian of Gaussian (LoG) with sigma values of 3.0 and 5.0, as well as square, square root, logarithm, exponential, gradient, and wavelet. Each unfiltered or filtered image type has 93 features, which can be divided into 6 subgroups according to different calculation algorithms: first-order, gray level co-occurrence matrix (GLCM), gray level size matrix (GLSZM), gray level run length matrix (GLRLM), neighborhood gray-tone difference matrix (NGTDM), and gray level dependence matrix (GLDM) with 18, 24, 16, 16, 5, 14 features for each, respectively. The bin width was set to 25, and the resampled pixel spacing was set to [3, 3, 3].

Concordance correlation coefficients (CCCs) were used to evaluate radiomics reproducibility between original and reconstructed images. The statistical significance of CCCs improvement was calculated with the paired t-test by comparing the CCCs of original thin images vs. reconstructed thin images and original thin images vs. original thick images.

Radiomics features with CCCs of 0.85 or higher were considered reproducible [6]. The numbers of reproducible radiomics features with CCC thresholds of 0.8, 0.85, and 0.9 were compared.

Section S2: Supplementary Tables

Table S1. Overview of the categories for the three whole-image level parameters for radiologist review.

	1	2	3	4
Realistic image appearance	Overall, not recognizable as CT	Overall, unrealistic, but generally recognizable as CT	Overall, realistic and only minor unrealistic areas	Can't tell whether fake or not
Consistency between slices	No Consistent slices	Only a few slices are consistent	The majority of slices are consistent	All slices are consistent
Anatomic correctness	Anatomic region not recognizable	Anatomic region is recognizable, but major parts of the images exhibit anatomic incorrectness	Only minor anatomic incorrectness	Anatomic features are correct

Table S2. Performance of 3DAD at different phases of the abdomen base dataset. A. non-contrast phase; B. Arterial phase; C. Portal-venous phase; and D. Delayed phase.

A. non-contrast phase

	Internal			External		
	MSE	PSNR	SSIM	MSE	PSNR	SSIM
2-slice[1]	22.350 (±33.723)	36.644 (±3.760)	0.973 (±0.020)	29.624 (±16.358)	33.993 (±1.918)	0.967 (±0.013)
3-slice	39.565 (±51.442)	33.456 (±3.002)	0.948 (±0.028)	50.337 (±26.812)	31.511 (±1.681)	0.944 (±0.019)
4-slice	60.749 (±73.932)	31.521 (±2.707)	0.923 (±0.035)	80.190 (±49.579)	29.664 (±1.782)	0.923 (±0.026)
5-slice	68.650 (±77.029)	30.740 (±2.475)	0.908 (±0.035)	86.493 (±45.699)	29.176 (±1.598)	0.914 (±0.026)
6-slice	87.930 (±93.911)	29.684 (±2.382)	0.892 (±0.040)	121.578 (±73.755)	27.911 (±1.763)	0.898 (±0.032)

B. Arterial phase

	Internal			External		
	MSE	PSNR	SSIM	MSE	PSNR	SSIM
2-slice	17.479 (±14.542)	36.934 (±3.062)	0.979 (±0.013)	27.602 (±17.450)	34.741 (±3.067)	0.975 (±0.013)
3-slice	33.514 (±21.008)	33.599 (±2.405)	0.957 (±0.019)	49.086 (±24.910)	31.824 (±2.355)	0.954 (±0.020)
4-slice	55.547 (±33.392)	31.407 (±2.242)	0.934 (±0.024)	79.103 (±40.606)	29.777 (±2.152)	0.935 (±0.025)
5-slice	62.497 (±30.940)	30.646 (±1.928)	0.921 (±0.025)	87.642 (±38.638)	29.158 (±1.912)	0.925 (±0.026)
6-slice	85.882 (±42.424)	29.317 (±1.875)	0.904 (±0.028)	123.368 (±59.924)	27.791 (±1.907)	0.910 (±0.030)

C. Portal-venous phase

	Internal			External		
	MSE[1]	PSNR[2]	SSIM[3]	MSE	PSNR	SSIM
2-slice[4]	18.981 (±17.699)	36.800 (±3.275)	0.977 (±0.015)	26.139 (±15.400)	35.000 (±3.109)	0.972 (±0.014)
3-slice	36.886 (±26.110)	33.343 (±2.611)	0.954 (±0.022)	47.944 (±23.402)	31.935 (±2.429)	0.948 (±0.021)
4-slice	57.386 (±36.615)	31.341 (±2.344)	0.929 (±0.026)	72.446 (±35.864)	30.155 (±2.152)	0.927 (±0.025)
5-slice	66.346 (±37.114)	30.491 (±2.105)	0.915 (±0.027)	81.374 (±35.117)	29.478 (±1.935)	0.916 (±0.027)
6-slice	87.436 (±47.153)	29.302 (±2.015)	0.898 (±0.030)	110.068 (±51.778)	28.249 (±1.888)	0.900 (±0.031)

D. Delayed phase

	Internal			External		
	MSE	PSNR	SSIM	MSE	PSNR	SSIM
2-slice	19.868 (±18.742)	36.657 (±3.422)	0.976 (±0.016)	36.054 (±15.178)	33.108 (±2.001)	0.964 (±0.009)
3-slice	37.038 (±28.439)	33.357 (±2.703)	0.952 (±0.023)	61.058 (±25.215)	30.697 (±1.917)	0.937 (±0.016)
4-slice	58.243 (±43.139)	31.308 (±2.384)	0.927 (±0.027)	91.254 (±39.850)	29.084 (±1.938)	0.915 (±0.021)
5-slice	66.574 (±46.252)	30.530 (±2.184)	0.913 (±0.028)	99.984 (±39.993)	28.533 (±1.820)	0.904 (±0.023)
6-slice	87.912 (±64.166)	29.364 (±2.102)	0.896 (±0.032)	129.252 (±55.038)	27.541 (±1.887)	0.890 (±0.027)

[1]n-slice: every n slice in the thin slices was compressed to one thick slice (n= 2,3,4,5,6).

Table S3. Performance of benchmarks at the portal-venous phase of the abdomen base dataset. A. Pix2pix; B. CycleGAN; C. 2.5-DAD[1].

A. Pix2Pix

	Internal			External		
	MSE	PSNR	SSIM	MSE	PSNR	SSIM
2-slice	31.602 (±22.506)	34.118 (±2.566)	0.964 (±0.017)	41.359 (±23.850)	32.846 (±2.678)	0.960 (±0.018)
3-slice	61.708 (±35.833)	30.939 (±2.123)	0.932 (±0.023)	73.303 (±31.850)	30.067 (±2.030)	0.932 (±0.023)
4-slice	103.683 (±58.215)	28.578 (±2.018)	0.899 (±0.030)	129.696 (±63.260)	27.543 (±1.932)	0.898 (±0.032)
5-slice	120.764 (±58.840)	27.826 (±1.842)	0.878 (±0.032)	141.692 (±56.319)	27.059 (±1.718)	0.885 (±0.031)
6-slice	158.662 (±74.940)	26.604 (±1.774)	0.853 (±0.036)	186.688 (±73.973)	25.848 (±1.623)	0.861 (±0.035)

B. CycleGAN

	Internal			External		
	MSE	PSNR	SSIM	MSE	PSNR	SSIM
2-slice	43.852 (±29.417)	32.594 (±2.314)	0.957 (±0.018)	55.649 (±27.799)	31.475 (±2.195)	0.954 (±0.017)
3-slice	99.333 (±57.523)	28.999 (±1.884)	0.915 (±0.026)	120.549 (±52.491)	28.170 (±1.734)	0.916 (±0.025)
4-slice	155.820 (±86.693)	27.151 (±1.863)	0.882 (±0.032)	195.438 (±86.877)	26.176 (±1.575)	0.883 (±0.029)
5-slice	187.429 (±96.301)	26.289 (±1.641)	0.863 (±0.034)	222.674 (±100.189)	25.618 (±1.531)	0.869 (±0.033)
6-slice	251.739 (±126.969)	25.065 (±1.559)	0.838 (±0.037)	307.782 (±145.569)	24.400 (±1.554)	0.846 (±0.038)

C. 2.5-DAD

	Internal			External		
	MSE	PSNR	SSIM	MSE	PSNR	SSIM
2-slice	42.496 (±23.277)	32.408 (±2.082)	0.955 (±0.016)	31.589 (±1.656)	31.589 (±1.656)	0.956 (±0.014)

3-slice	71.245 (±36.833)	30.153 (±1.948)	0.924 (±0.027)	86.667 (±33.790)	29.175 (±1.596)	0.926 (±0.022)
4-slice	100.992 (±48.376)	28.597 (±1.793)	0.899 (±0.031)	122.229 (±47.513)	27.688 (±1.511)	0.902 (±0.026)
5-slice	126.168 (±59.569	27.634 (±1.751)	0.880 (±0.033)	154.385 (±61.820)	26.722 (±1.498)	0.883 (±0.028)
6-slice	162.433 (±73.315)	26.557 (±1.657)	0.851 (±0.031)	196.269 (±78.646)	25.728 (±1.456)	0.843 (±0.025)

[1] 2.5-DAD indicated the adversarial diffusion model focuses on synthesizing thin-slice images from each thick-slice independently, for n=2, 3, 4, 5, and 6, respectively. For example, if n=5, 2.5-DAD compresses 5 thin-slices into one thick-slice and systhesizes 5 thin-slices from the compressed thick-slice, without considering the volume information of the other thin-slices.

Table S4. AUCs for the diagnosis of HCC were compared between synthetic and real thin-slice CT for the abdomen base dataset using ST3DCN. No significant difference was found in the internal dataset at either the observation or patient level. For the external dataset, significant differences were found for the 4-, 5-, 6-slice scenarios at the observation level and for the 4-, 6-slice scenarios at the patient level. The paired t-test was used.

	Internal				External			
	Observation		Patient		Observation		Patient	
	AUC	P-value	AUC	P-value	AUC	P-value	AUC	P-value
Original	0.933	-	0.923	-	0.889	-	0.846	-
2-slice	0.93	0.216	0.92	0.322	0.891	0.430	0.848	0.395
3-slice	0.93	0.236	0.921	0.382	0.892	0.228	0.849	0.431
4-slice	0.927	0.062	0.918	0.156	0.897	0.009	0.855	0.027
5-slice	0.929	0.109	0.919	0.186	0.896	0.028	0.854	0.055
6-slice	0.928	0.078	0.919	0.168	0.896	0.029	0.855	0.035

Table S5. CCC per image filter types between real and synthetic thin-slice CT for fine-tuning testing data. Data are presented with CCC ± standard deviation. CCC: concordance correlation coefficient. [1] For those p-values missed, represents $p < 0.001$, compared between real thin-slice vs. synthetic thin-slice and real thin-slice vs. real thick-slice CT images.

	Non-Contrast Phase	Arterial Phase	Portal Venous Phase	Delayed Phase
Total (N = 1488)	0.700 ± 0.244 (0.606)	0.802 ± 0.192 [1]	0.769 ± 0.230[1]	0.810 ± 0.199 [1]
Original (N = 93)	0.631 ± 0.218 (0.620)	0.773 ± 0.209 [1]	0.754 ± 0.201 [1]	0.782 ± 0.197 [1]
LoG (σ = 3) (N = 93)	0.757 ± 0.209 [1]	0.891 ± 0.127 [1]	0.908 ± 0.095 [1]	0.930 ± 0.103 [1]
LoG (σ = 5) (N = 93)	0.872 ± 0.141 (0.690)	0.933 ± 0.114 [1]	0.953 ± 0.072 [1]	0.959 ± 0.071 [1]
Square (N = 93)	0.641 ± 0.206 (0.524)	0.830 ± 0.164 [1]	0.793 ± 0.167 [1]	0.754 ± 0.174 [1]
Squareroot (N = 93)	0.576 ± 0.237 (0.026)	0.749 ± 0.204 [1]	0.659 ± 0.278 [1]	0.767 ± 0.194 [1]
Logarithm (N = 93)	0.541 ± 0.264 [1]	0.660 ± 0.253 [1]	0.571 ± 0.316 [1]	0.738 ± 0.196 [1]
Exponential (N = 93)	0.729 ± 0.236 (0.052)	0.904 ± 0.111 [1]	0.869 ± 0.146 [1]	0.816 ± 0.199 (0.017)
Gradient (N = 93)	0.863 ± 0.116 (0.645)	0.879 ± 0.105 [1]	0.859 ± 0.156 [1]	0.934 ± 0.083 [1]
Wavelet-LLH (N = 93)	0.834 ± 0.181 [1]	0.842 ± 0.125 [1]	0.857 ± 0.157 [1]	0.825 ± 0.139 (0.048)
Wavelet-LHL (N = 93)	0.867 ± 0.128 [1]	0.852 ± 0.139 [1]	0.843 ± 0.132 [1]	0.895 ± 0.112 [1]
Wavelet-LHH (N = 93)	0.765 ± 0.196 (0.9)	0.753 ± 0.172 [1]	0.746 ± 0.154 [1]	0.670 ± 0.244 (0.045)
Wavelet-HLL (N = 93)	0.612 ± 0.210 (0.117)	0.795 ± 0.173 [1]	0.836 ± 0.130 [1]	0.841 ± 0.166 [1]
Wavelet-HLH (N = 93)	0.611 ± 0.274 [1]	0.663 ± 0.210 [1]	0.596 ± 0.258 [1]	0.737 ± 0.205 [1]
Wavelet-HHL (N = 93)	0.608 ± 0.236 [1]	0.742 ± 0.183 [1]	0.566 ± 0.233 [1]	0.748 ± 0.200 [1]
Wavelet-HHH (N = 93)	0.501 ± 0.305 [1]	0.646 ± 0.212 [1]	0.585 ± 0.246 [1]	0.603 ± 0.247 [1]
Wavelet-LLL (N = 93)	0.791 ± 0.143 [1]	0.924 ± 0.105 [1]	0.913 ± 0.095 [1]	0.953 ± 0.067 [1]

Table S6. CCC per image filter types between real thin-slice and real thick-slice (5mm) CT images for fine-tuning testing data. Data are presented with CCC ± standard deviation. CCC: concordance correlation coefficient.

	Non-Contrast Phase	Arterial Phase	Portal Venous Phase	Delayed Phase
Total (N = 1488)	0.702 ± 0.256	0.704 ± 0.246	0.664 ± 0.275	0.702 ± 0.244
Original (N = 93)	0.620 ± 0.268	0.656 ± 0.223	0.592 ± 0.270	0.628 ± 0.231
LoG (σ = 3) (N = 93)	0.824 ± 0.184	0.817 ± 0.188	0.801 ± 0.181	0.845 ± 0.122
LoG (σ = 5) (N = 93)	0.874 ± 0.148	0.850 ± 0.186	0.851 ± 0.166	0.862 ± 0.137
Square (N = 93)	0.629 ± 0.253	0.789 ± 0.184	0.737 ± 0.176	0.681 ± 0.186
Squareroot (N = 93)	0.637 ± 0.251	0.528 ± 0.272	0.489 ± 0.338	0.544 ± 0.255
Logarithm (N = 93)	0.688 ± 0.230	0.491 ± 0.300	0.460 ± 0.352	0.481 ± 0.288
Exponential (N = 93)	0.755 ± 0.218	0.856 ± 0.169	0.801 ± 0.184	0.795 ± 0.227
Gradient (N = 93)	0.858 ± 0.149	0.795 ± 0.146	0.638 ± 0.239	0.825 ± 0.159
Wavelet-LLH (N = 93)	0.866 ± 0.136	0.790 ± 0.163	0.818 ± 0.140	0.800 ± 0.171
Wavelet-LHL (N = 93)	0.834 ± 0.147	0.801 ± 0.157	0.809 ± 0.137	0.818 ± 0.138
Wavelet-LHH (N = 93)	0.766 ± 0.188	0.716 ± 0.198	0.674 ± 0.207	0.639 ± 0.256
Wavelet-HLL (N = 93)	0.642 ± 0.201	0.741 ± 0.185	0.770 ± 0.191	0.748 ± 0.216
Wavelet-HLH (N = 93)	0.552 ± 0.263	0.510 ± 0.267	0.485 ± 0.269	0.653 ± 0.230
Wavelet-HHL (N = 93)	0.439 ± 0.257	0.585 ± 0.234	0.483 ± 0.253	0.618 ± 0.230
Wavelet-HHH (N = 93)	0.422 ± 0.285	0.535 ± 0.243	0.460 ± 0.284	0.499 ± 0.264
Wavelet-LLL (N = 93)	0.831 ± 0.113	0.809 ± 0.173	0.763 ± 0.225	0.790 ± 0.184

Section S3: Supplementary Figures

Figure S1. CCCs heatmap for 1488 radiomics features for all image types. The color bar (ranging from 0 to 1) indicates the CCC values. Deeper green represents higher CCC values (closer to 1), while deeper magenta indicates lower CCC values (closer to 0).

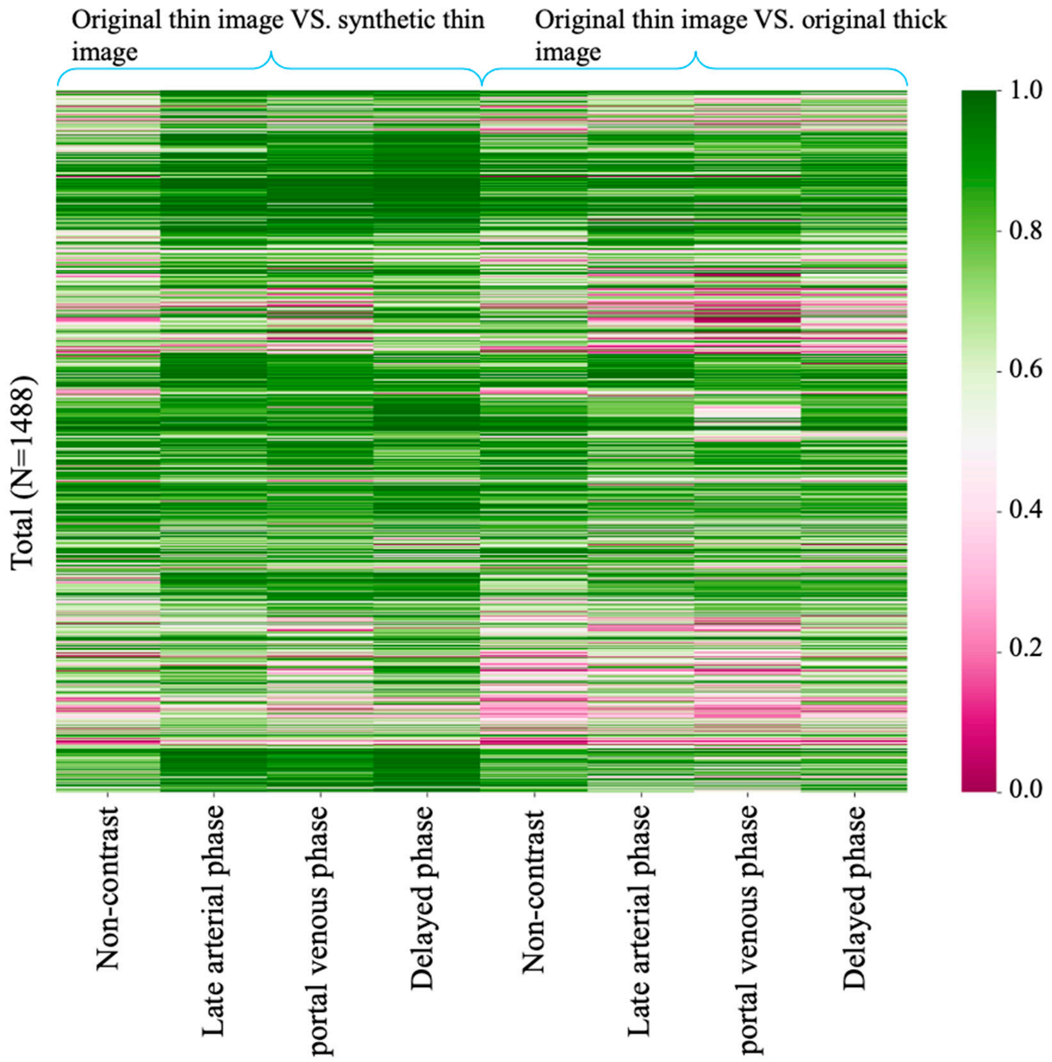

Figure S2. LIRADS diagnosis between real and synthetic thin-slice CT images.

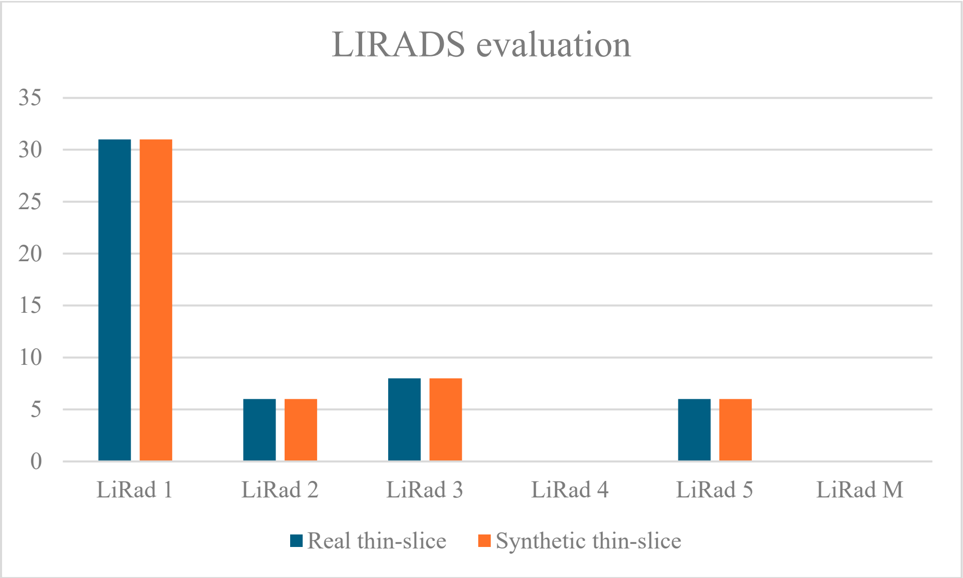

References

1. Yu, P.L.H.; Chiu, K.W.-H.; Lu, J.; Lui, G.C.; Zhou, J.; Cheng, H.-M.; Mao, X.; Wu, J.; Shen, X.-P.; Kwok, K.M. Application of a deep learning algorithm for the diagnosis of HCC. *JHEP Reports* **2025**, *7*, 101219.
2. Ibanez, L.; Schroeder, W.; Ng, L.; Cates, J. The ITK software guide. **2003**.
3. Özbey, M.; Dalmaz, O.; Dar, S.U.; Bedel, H.A.; Özturk, Ş.; Güngör, A.; Cukur, T. Unsupervised medical image translation with adversarial diffusion models. *IEEE Transactions on Medical Imaging* **2023**, *42*, 3524-3539.
4. Wang, G.; Duan, Q.; Shen, T.; Zhang, S. SenseCare: a research platform for medical image informatics and interactive 3D visualization. *Frontiers in Radiology* **2024**, *4*, 1460889.
5. Van Griethuysen, J.J.; Fedorov, A.; Parmar, C.; Hosny, A.; Aucoin, N.; Narayan, V.; Beets-Tan, R.G.; Fillion-Robin, J.-C.; Pieper, S.; Aerts, H.J. Computational radiomics system to decode the radiographic phenotype. *Cancer research* **2017**, *77*, e104-e107.
6. Lee, H.; Chang, W.; Kim, H.Y.; Sung, P.; Cho, J.; Lee, Y.J.; Kim, Y.H. Improving radiomics reproducibility using deep learning-based image conversion of CT reconstruction algorithms in hepatocellular carcinoma patients. *European Radiology* **2024**, *34*, 2036-2047.
